# Laparoscopic Resection of a Cecal Schwannoma Presenting With Intussusception After a Seven-Year Follow-Up: A Case Report

**DOI:** 10.7759/cureus.87355

**Published:** 2025-07-05

**Authors:** Toru Takahashi, Kimihiko Ueno, Maria Takeda

**Affiliations:** 1 Division of Hepato-Biliary-Pancreatic Surgery, Kobe University Graduate School of Medicine, Kobe, JPN; 2 Department of Surgery, National Hospital Organization Kobe Medical Center, Kobe, JPN

**Keywords:** case report, cecal schwannoma, colonic schwannoma, intussusception, laparoscopic resection

## Abstract

An 88-year-old woman was diagnosed with a submucosal tumor of the cecum during a lower gastrointestinal endoscopy performed seven years earlier and had since been under regular follow-up. In August 2021, she began experiencing left-sided abdominal pain and bloating. A computed tomography (CT) scan conducted in September revealed a target sign in the ascending colon, with the tumor identified as the lead point of intussusception. She was referred to our department, where laparoscopic ileocecal resection was performed in November. Histopathological examination confirmed the diagnosis of schwannoma. Primary schwannomas of the colon are extremely rare, and cases originating in the cecum are particularly uncommon. We herein report a rare case of laparoscopically resected cecal schwannoma that developed intussusception after a prolonged follow-up period.

## Introduction

Schwannomas are tumors that originate from Schwann cells and are commonly found in superficial areas, such as the head, neck, and extremities. However, those arising in the gastrointestinal tract are relatively rare, and primary schwannomas of the colon are exceedingly uncommon [1]. Herein, we report a rare case of cecal schwannoma that developed intussusception after a prolonged follow-up period, successfully treated with laparoscopic surgery.

Primary schwannomas of the colon are extremely rare, with only a limited number of cases reported in the literature. Among them, occurrences in the cecum are particularly scarce, highlighting the unusual nature of the present case [2].

In this report, we present a case of cecal schwannoma that caused intussusception after a prolonged follow-up period, which was successfully treated with laparoscopic resection. The case underscores the importance of considering surgical intervention even in asymptomatic submucosal tumors when long-term follow-up reveals progressive clinical manifestations.

## Case presentation

An 88-year-old woman presented with complaints of left-sided abdominal pain and abdominal distension. Seven years earlier, a submucosal tumor of the cecum had been detected during a lower gastrointestinal endoscopy, and she had been under regular follow-up. In August 2021, she began experiencing new abdominal symptoms. A computed tomography (CT) scan performed in September revealed that the submucosal tumor had formed a target sign in the ascending colon, indicating intussusception with the tumor as the lead point. She was subsequently referred to our department for further evaluation and management.

Her past medical history included hypertension, gastroesophageal reflux disease, chronic constipation, cystitis, glaucoma, and a history of *Helicobacter pylori* eradication. There was no notable family history. On admission, her height was 150.5 cm, her weight was 42.5 kg, and her body mass index (BMI) was 18.8. The abdomen was flat and soft, with no tenderness, but a palpable mass was detected in the right lower quadrant.

The patient presented with mild inflammation and anemia, as well as normal tumor marker values (Table 1), which were consistent with her clinical presentation.

**Table 1:** Laboratory values on the first day of admission. WBC: white blood cell; RBC: red blood cell; BUN: blood urea nitrogen; eGFR: estimated glomerular filtration rate; ALT: alanine transaminase; AST: aspartate transaminase; ALP: alkaline phosphatase; CRP: C-reactive protein; CEA: carcinoembryonic antigen; CA19-9: carbohydrate antigen 19-9.

Component	Value	Normal Range
WBC	4.5 k/mm^3^	3.5–9.0
Platelets	297 k/mm^3^	150–400
RBC	3.44 m/nm^3^	3.8–5.1
Hemoglobin	9.8 g/dL	11.5–15.0
BUN	10 mg/dL	8–20
Creatinine	0.66 mg/dL	0.5–0.9
eGFR	62 mL/min/1.73 m^2^	>60
ALT	9 IU/L	5–40
AST	15 IU/L	5–35
ALP	59 IU/L	35–100
Total bilirubin	0.36 mg/dL	0.2–1.2
Total protein	6.4 g/dL	6.5–8.0
Albumin	3.1 g/dL	3.5–5.0
CRP	1.52 mg/dL	<0.3
CEA	1.4 ng/mL	<5.0
CA19-9	7.3 U/mL	<37

**Figure 1:**
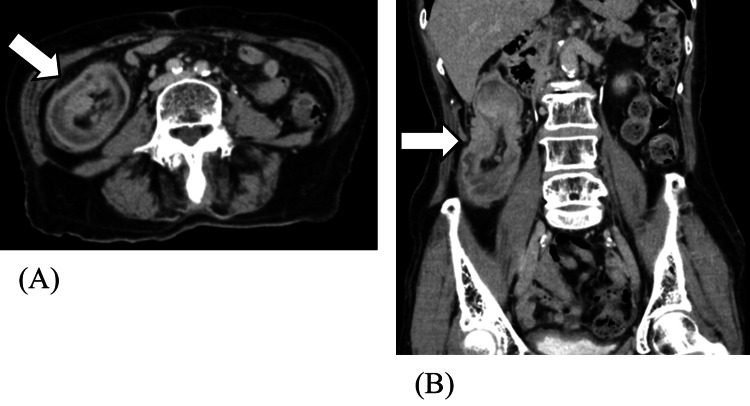
Abdominal contrast-enhanced CT findings at presentation. Axial (A) and coronal (B) slice CT scans of the abdomen show that a 41-mm soft tissue mass with mild enhancement is seen in the cecum (arrow), located at the level of the inferior hepatic margin. A concentric ring appearance (target sign) is visible in the ascending colon, consistent with intussusception, with the tumor as the lead point.

Contrast-enhanced abdominal CT demonstrated a 41-mm mass in the cecum with mild enhancement, visualized at the level of the inferior hepatic margin. A concentric ring pattern (target sign) was noted in the ascending colon, consistent with intussusception led by the tumor. No evidence of intestinal obstruction was observed (Figure 1).

**Figure 2:**
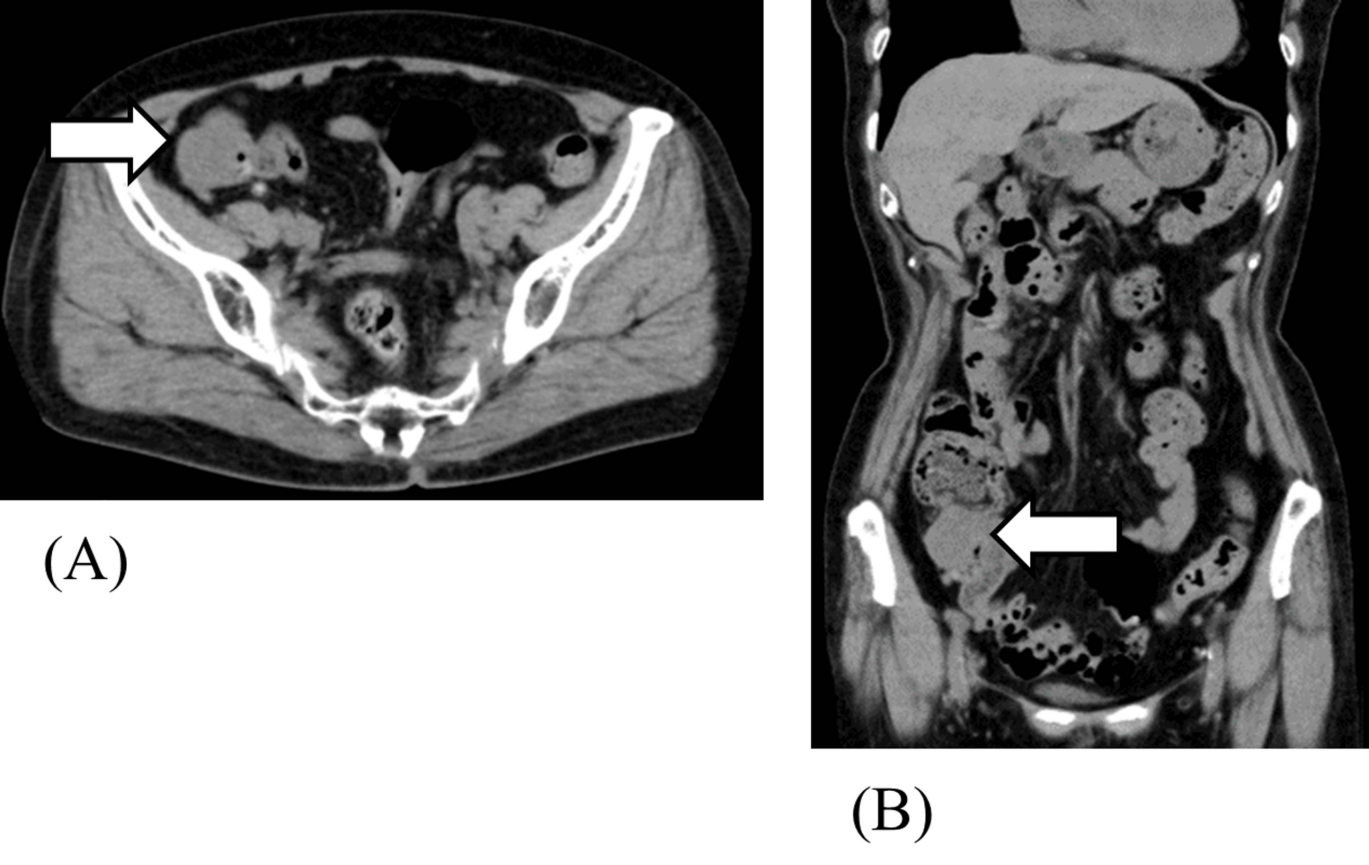
Abdominal plain CT findings obtained seven years earlier. Axial (A) and coronal (B) slice CT scans of the abdomen show that a known 3-cm submucosal mass is observed at the ileocecal region (arrow). No target sign is present at that time.

A review of a plain abdominal CT scan taken seven years earlier revealed a known 3-cm mass in the ileocecal region, without any target sign (Figure 2). An abdominal CT scan in 2018 showed the tumor remained 30 mm (unchanged from 2014); however, a CT in 2021 revealed enlargement to 41 mm.

Intraoperative findings via laparoscopy revealed serosal retraction of the cecum caused by the tumor. Given the possibility of malignancy, an ileocecal resection including the tumor was performed along with D3 lymph node dissection. Although the tumor had been followed as a submucosal lesion for several years, a definitive preoperative diagnosis was not achieved. Based on intraoperative findings such as firm consistency and serosal indentation, differential diagnoses, including gastrointestinal stromal tumor (GIST), adenocarcinoma, or lymphoma, could not be excluded. Therefore, D3 lymphadenectomy was carried out to ensure oncological completeness in the event of a malignant pathology. Reconstruction was achieved by a functional end-to-end anastomosis using a linear stapler between the ileum and the ascending colon.

**Figure 3:**
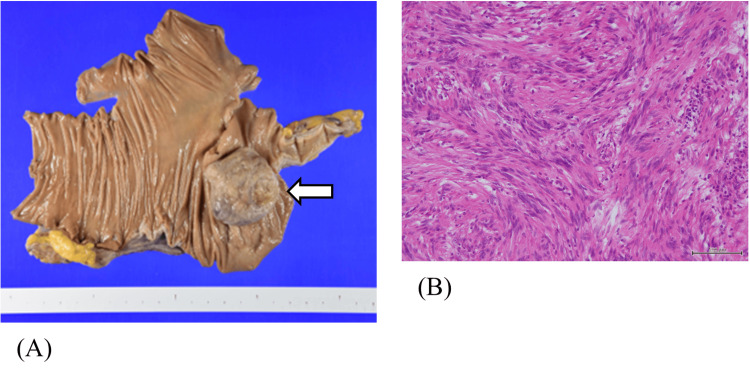
Pathological findings of the resected specimen. (A) Macroscopic image showing a Type 1 tumor measuring 48 × 42 mm in the cecum. (B) Hematoxylin and eosin (H&E) staining demonstrates dense proliferation of spindle- and oval-shaped cells in the submucosa.

**Figure 4:**
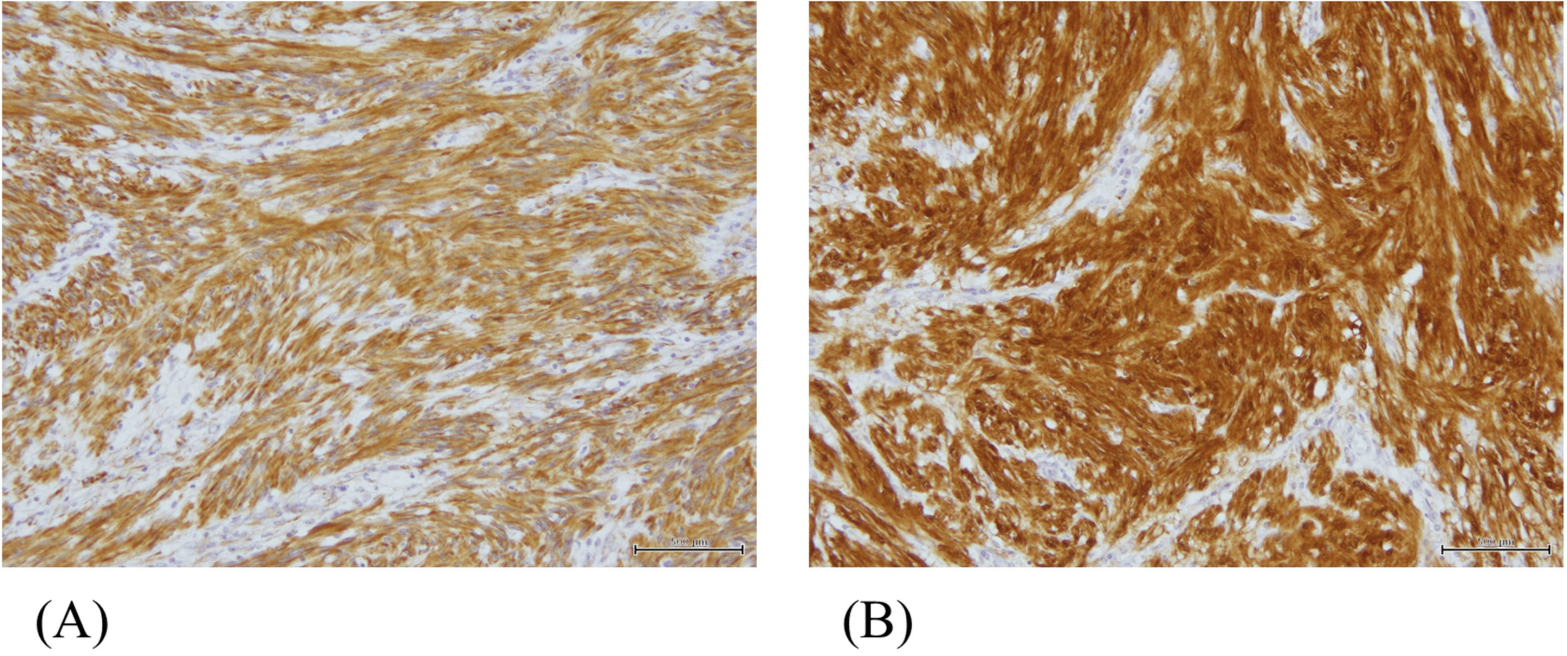
Pathological findings of the resected specimen. Immunohistochemical staining shows strong positivity for vimentin (A) and S-100 protein (B).

**Figure 5:**
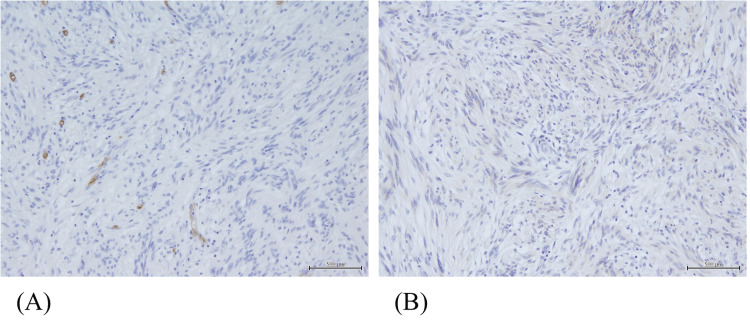
Pathological findings of the resected specimen. Negative staining for CD34 (A) and CD117/c-kit (B), supporting the diagnosis of schwannoma.

Gross pathology revealed a Type 1 tumor measuring 48 × 42 mm in the cecum. Hematoxylin and eosin (H&E) staining showed dense proliferation of spindle-shaped and oval cells in the submucosal layer. Immunohistochemically, the tumor was strongly positive for vimentin and S-100 protein, while negative for CD34 and CD117 (c-kit), consistent with the diagnosis of schwannoma (Figures 3-5). The tumor infiltrated the muscularis mucosae and subserosa. Focal necrosis, nuclear pleomorphism, and cellular atypia were also observed. No vascular or perineural invasion was noted, and all resection margins (oral, anal, and mesenteric) were negative. Lymph nodes showed reactive hyperplasia without evidence of metastasis.

Postoperatively, the patient developed small bowel obstruction. Conservative treatment was initially attempted but proved ineffective, necessitating surgical intervention with ileus release surgery. She was discharged from the hospital on postoperative day 52.

## Discussion

Schwannomas are tumors derived from Schwann cells and typically arise in peripheral nerve tissues. Their occurrence in the gastrointestinal tract is rare, with the majority found in the stomach and small intestine. Colonic involvement, particularly in the lower gastrointestinal tract, is exceedingly uncommon [1]. According to a report by Misao et al., only 97 cases of colonic schwannomas were documented in Japan between 1940 and 2018 [2]. Patient ages ranged from 12 to 88 years (mean, 59.9 years), with no significant difference in sex distribution. Among these, the rectum was the most frequently affected site, followed by the ascending colon, transverse colon, sigmoid colon, descending colon, cecum, and appendix. Only seven cases involved the cecum, underscoring the rarity of the present case. Colonic schwannomas typically present with nonspecific clinical symptoms and are often incidentally discovered during routine screening in asymptomatic individuals [2]. Endoscopic and barium enema examinations do not yield characteristic findings, and preoperative biopsy is often inconclusive. While an FDG-PET (fluorodeoxyglucose positron emission tomography) scan is sometimes used to differentiate benign from malignant lesions, fluorodeoxyglucose uptake is frequently observed even in histologically benign schwannomas [3,4]. Beaulieu et al. reported FDG uptake in eight of nine cases of colonic schwannoma, despite all being pathologically benign [3]. Consequently, accurate preoperative diagnosis remains extremely difficult. In the present case, a definitive diagnosis of schwannoma was only achieved through histopathological examination of the resected specimen.

In the present case, a submucosal tumor of the cecum was initially identified seven years earlier. Due to its stable size and asymptomatic nature, the tumor was managed conservatively with regular follow-up. However, after seven years, the patient developed intussusception, and the tumor had enlarged to approximately 4 cm. Schwannomas of the colon may gradually increase in size over time and eventually lead to symptomatic presentations [1]. Therefore, it is generally recommended that such tumors be resected upon detection, even if they are asymptomatic. In this case, the lesion had been presumed benign and was followed without intervention, but surgical treatment was ultimately required when clinical symptoms appeared.

In Japan, only three prior cases of cecal schwannoma associated with intussusception have been reported [2,5], making the present case the fourth. In previously reported cases, the diagnosis of intussusception was made based on palpable masses or radiological findings. However, none involved long-term follow-up preceding the onset of intussusception. Notably, in our case, intussusception developed despite minimal changes in tumor size over the follow-up period. This highlights the importance of considering surgical resection as part of the management strategy, even in cases where the tumor appears stable, to prevent acute complications such as intussusception.

Schwannomas are generally considered benign tumors, and malignant transformation is extremely rare, with the exception of rare variants such as melanotic schwannoma [6-8]. Even in cases where submucosal invasion, necrosis, or nuclear atypia is observed histologically, schwannoma should be distinguished from malignant peripheral nerve sheath tumors (MPNST), which are regarded as a separate entity [9]. Although lymphadenectomy is not usually required for benign schwannomas, in this case, a definitive preoperative diagnosis was not available. Intraoperative characteristics such as serosal retraction and firm tumor consistency raised suspicion for malignancies such as GIST or adenocarcinoma. As a result, D3 lymph node dissection was performed to adhere to oncologic principles in the context of diagnostic uncertainty. Ultimately, the tumor was pathologically diagnosed as a schwannoma. Given the invasive features into the muscularis mucosae and subserosa, focal necrosis, and marked nuclear pleomorphism observed histologically, the decision to perform an oncologically appropriate resection, including lymphadenectomy, was deemed justified.

In this case, histopathological evaluation demonstrated dense proliferation of spindle- and oval-shaped cells in the submucosal layer, with strong immunoreactivity for S-100 protein and negativity for CD34 and c-kit, confirming the diagnosis of schwannoma. Although the tumor exhibited minimal growth during the follow-up period, the patient eventually developed intussusception, necessitating surgical intervention. This case suggests that even asymptomatic cecal schwannomas have the potential to cause acute complications such as intussusception. Therefore, early surgical resection should be considered as a proactive strategy, even when the tumor appears stable and benign.

## Conclusions

We experienced a rare case of cecal schwannoma that developed intussusception after a seven-year period of observation and required surgical intervention. Although cecal schwannomas may remain asymptomatic for extended periods, they have the potential to cause acute complications such as intussusception. Therefore, surgical resection should be considered in the management of such tumors, even when asymptomatic at the time of diagnosis.
